# The Applications of Nanopore Sequencing Technology in Pathogenic Microorganism Detection

**DOI:** 10.1155/2020/6675206

**Published:** 2020-12-31

**Authors:** Xiaojian Zhu, Shanshan Yan, Fenghua Yuan, Shaogui Wan

**Affiliations:** ^1^Center for Molecular Pathology, Department of Basic Medicine, Gannan Medical University, Ganzhou 341000, China; ^2^Key Laboratory of Prevention and Treatment of Cardiovascular and Cerebrovascular Diseases of Ministry of Education, Gannan Medical University, Ganzhou 341000, China; ^3^Department of Publication Health and Health Management, Gannan Medical University, Ganzhou 341000, China

## Abstract

Infectious diseases are major threats to human health and lead to a serious public health burden. The emergence of new pathogens and the mutation of known pathogens challenge our ability to diagnose and control infectious diseases. Nanopore sequencing technology exhibited versatile applications in pathogenic microorganism detection due to its flexible data throughput. This review article introduced the applications of nanopore sequencing in clinical microbiology and infectious diseases management, including the monitoring of emerging infectious diseases outbreak, identification of pathogen drug resistance, and disease-related microbial communities characterization.

Infectious disease leads to significant health care burden in the world. Although we have made great progress in the prevention and control of infectious diseases [1], the emerging of new pathogen and reemerging of classical pathogens epidemics still pose serious threats to human health. The number of cases of respiratory infections and *tuberculosis* in the world in 2017 was as high as 17.9 billion [2]. At the same time, new pathogens are still emerging, including the outbreak of SARS-CoV in 2002–2003 [3, 4], Middle East respiratory syndrome coronavirus (MERS-CoV) in 2012 [5], Ebola virus in West Africa [6], and the recent global epidemic of SARS-COV-2 [7], with 30-day hospital mortality as high as 20% [8]. Diagnosis and control of infectious diseases is still a protracted war.

Characterization of infectious-causing microorganisms is essential for surveillance, treatment, and control of infectious diseases. Difficulty to isolate and culture microorganisms, complex of community composition, and high level of genomic plasticity impede the understanding of infectious diseases [9]. Microbial detection based on traditional culture method is still regarded as the gold standard in clinical. However, it is time-consuming and is limited to cultivable pathogens [10]. The method of RT-qPCR is rapid, specific, and economic; however, it is limited to known pathogens, resulting in missed diagnosis of unknown pathogens [11]. Furthermore, it is unable to detect the mutation of the pathogen (Table 1). The plasticity of pathogen genome endows them with the ability of mutation adaptation under environmental pressure (antibiotic exposure, etc.) [22], and these mutations are often disadvantageous to the host, which may lead to the increase in antibiotic resistance or virulence [23], thus becoming a threat to antibacterial chemotherapy.

The rise of DNA sequencing technology provides an effective way to better understand infectious diseases. Over the past decades, next-generation sequencing (NGS) has become popular in clinical microbiology research and clinic settings. The method of metagenomic NGS has the ability to accurately detect all microorganisms in the sample without culturing within 24 hours. With the development of NGS technology, the detection cost is gradually decreased [24]. However, the detection result could not be analyzed immediately because the sequencing principle of NGS is based on the assemble of short reads. The bioinformatics analysis could be done only after the complete of the sequencing. Due to the short reads, it is difficult to parse the complex genome structure of microorganisms which usually contain many duplicates [25].

In recent years, innovations in third-generation sequencing technology, represented by Oxford Nanopore Technologies (ONT) and SMRT Pacific Biosciences (PacBio), have been able to produce longer reads in real time [26, 27]. Its extralong reads allow it to assemble the microbial genome in its entirety. The ONT is a portable equipment which allows the identification of pathogenic microorganisms on-site with lower cost per run and is more convenient to clinical laboratories [20]. Compared to NGS, the turnaround time of nanopore is shorter (Table 1). From sample collection to data acquisition, it takes only a few hours [28, 29]. At present, nanopore sequencing platforms have appeared in many clinical microbiology laboratories. Here, we highlight the applications of nanopore sequencing technique in infectious diseases, including monitoring of emerging infectious diseases outbreak, identification of pathogen drug resistance, and disease-related microbial communities characterization.

## 1. Rapid Identification of Pathogenic Microorganisms

Rapid and accurate identification of pathogenic microorganisms is a key link in the diagnosis and treatment of clinical infectious diseases. Currently, culture is still the main method for pathogen detection in clinic. However, it takes a long turnaround time, often 5–7 days [12]. Although NGS platforms such as Illumina have been used to track infectious diseases in hospitals, the inability to detect on-site is one of the drawbacks. The nanopore sequencing technology based on ONT platform allows the identification of pathogenic microorganisms on-site and is suitable for a wide range of clinical samples, including difficult-to-cultivate pathogens and opportunistic pathogens [30–33] (Table 2).

Due to the diversity of pathogens that can cause infection, the etiological diagnosis of infectious diseases is challenging. Several studies have shown that, in the detection of culture-positive samples, nanopore sequencing results show a high degree of consistency with microbiological culture results [30, 31, 36]. At the same time, information on possible bacterial pathogens was also obtained in culture-negative samples [36]. In addition, the composition of fungal communities can also be fully characterized by nanopore-based metagenomics [32]. Because of its speed and sensitivity, this method may be more effective than conventional diagnostic tests in the diagnosis of infectious diseases, and this may provide valuable information for further tracking of pathogens missed or undetectable by conventional microbial culture. Importantly, for critically ill patients, nanopore sequencing can provide the required results within feasible time range of clinical diagnosis [36, 37], which can better serve the clinic.

The emergence of new pathogens and mutations in existing pathogens leading to unpredictable outbreaks will continue to pose challenges to public health [38]. Metagenome sequencing based on ONT is a powerful tool for tracking disease outbreaks and transmission dynamics. Nanopore technology has shown its stability in the effective identification and monitoring of zoonotic infections such as Usutu virus [21], Ebola virus [20], Zika virus [39], and yellow fever virus [40]. At the same time, without relying on expensive facilities and instruments, the nanopore sequencing platform allows it to operate in a variety of extreme environments [41, 42]. In particular, the pocket sequencer MinION launched by ONT is more portable and cheaper for on-site sequencing and genetic analysis of outbreaks. More recently, the outbreak of COVID-19 has captured global attention. In January of this year, the *SARS-COV-2* (WH-Human_1) genome sequence was published for the first time in China and shared globally [43]. Later, Wang et al. innovatively developed Nanopore Targeted Sequencing (NTS) detection method [44], which obviously promoted the confirmation of infected patients. At the same time, the device detects outbreaks in real time, allowing researchers to track how the disease spreads and the rapid evolution of infectious agents [20]. This has brought great benefits to the prevention and control of the epidemic. Among them, the Chinese Center for Disease Control uses 3 sequencing technologies, including nanopore sequencing. Six patients' (COVID-19) samples were used for genome-wide phylogenetic analysis which describes the origin of the virus and, at the same time, through the homology model infer that the virus may have human angiotensin-converting enzyme 2 (ACE2) receptor binding properties [45]. The combination of nanopore sequencing data with epidemiological findings can help identify infected populations and guide outbreak control decisions. The discovery of this important information made a great contribution in tracing the source of the outbreak and in the management of patients.

Another advantage of nanopore sequencing is that the direct RNA sequencing could be realized. RNA of many viruses, including all influenza viruses and polio, was used as a repository for genetic information [29, 46]. In 2015, the RNA virus was sequenced for the first time using nanopore sequencing, and in just four hours, a reliable sketch of the influenza genome was produced [47]. What is more surprising, the influenza virus genome has been reported to be detected in the form of natural RNA [48]. Previous RNA sequencing of almost all depended on RNA reverse transcription or amplification [49]. Nanopore technology provides a more convenient method for sequencing RNA molecules in natural situation. Furthermore, direct sequencing of RNA molecules can help to reveal mysterious epigenetic RNA modifications, and some modifications may be the source of pathogen resistance [50]. It is not hard to imagine the potential for such portable sequencing tools, without dedicated devices and high-end computing resources, to detect RNA viruses directly from clinical samples during a viral pandemic or outbreak. This allows researchers to conduct field investigations and obtain clinically useful information in a very short period of time [51].

In addition, another typical application is the analysis of outbreak-related isolates using nanopores to reveal evolutionary history and indicate mutation information. Currently, the sensitivity of previous diagnostic methods using the *M* gene has been reduced due to the new gene mutation in the H1N1 and H3N2 virus [52]. The whole genome of clinical samples of influenza A virus was sequenced by nanopores, the relevant isolates were analyzed, and the mutation information was indicated [53]. Later, two genes PB2 and NS were identified and selected as new diagnostic targets for influenza A virus detection. At the same time, due to the interaction between pathogen and host, it shows the ability to adapt to mutation frequently [22]. It is also necessary to continuously update genomic information and screen for possible outbreak-related event strains [54]. Moreover, by analyzing the genome sequence of pathogens, candidate genes of vaccine antigens can be screened out, which can contribute to the development and design of subsequent vaccines. Overall, nanopore sequencing can monitor outbreaks and provide increasingly accurate and timely guidance for outbreak management, prevention, and control and for the evolution research.

## 2. Detection of Antibiotic Resistance

Identifying key characteristics of pathogens such as antimicrobial resistance (AMR) and pathogenicity is critical for therapeutic implications [55]. Repeated sequences of genomes and mobile genetic elements such as plasmids often contain important drug resistance and pathogenicity elements, yet such complex genomes are difficult to assemble in their integrity [56]. Nowadays, with the prevalence of NGS, there is still a gap in the understanding of the virulence and AMR of bacteria. In the early study, the potential of MinION to resolve bacterial antibiotic resistance islands was described although the accuracy rate was only 72% [57]. This may be due to the low coverage of the genome in the early operation of MinION. With the continuous upgrading and improvement of ONT technology and the upgrading of chips, the detection sensitivity of DNA single bases has been greatly improved [58]. In 2017, MinION alone was used to detect antibiotic resistance genes in three clinical isolates of *Klebsiella pneumoniae*, with an assembly accuracy of 99% [59]. At the same time, with the improvement of sequencing depth, the accuracy of nanopore assembly will be improved further. ONT released a new R10 chip in July 2019 that claims to have a Q50 level of common sequence accuracy for its nanopores—equivalent to one error per 100,000 bases, or 99.999 percent accuracy. In general, nanopore sequencing has great advantages in rapid identification of pathogens and analysis of antimicrobial resistance.

It is important to note that mobile genetic elements (MGEs) also often carry a large amount of resistance information, and the capture and analysis of these MGEs can explain and refine antibiotic resistance phenotypes in clinical isolates [60]. However, the widely used NGS assembly of MGEs is often highly fragmented and can result in omissions [61]. This impedes the proper identification of plasmids, phages, and virulence factors. However, the long reading length of nanopore sequencing shows great advantage in this aspect. In one study, three plasmids from *Klebsiella pneumoniae* were isolated, and AMR genes were obtained by nanopore sequencing alone, with an assembly accuracy of about 99% [59]. More significantly, the study was sufficient to describe antibiotic resistance information on plasmid DNA at a low reading depth in as little as 20 minutes, balancing the relationship between turnaround time and accuracy. Shiga toxin-producing *E. coli* (STEC) is a highly pathogenic hemorrhagic pathogen with high incidence rate and high lethality. The genome is rich in plasmids, phages, and virulence genes [62, 63]. Recently, 3 STEC genomes were sequenced by Illumina and MinION platforms. The MinION data provided genomic location information for 20 phages, while in Illumina sequencing data, individual phages could not be reconstructed [64]. Meanwhile, MinION sequencing found that one strain carried multiple AMR genes, all in a tiny plasmid [64]. Equally significant, the hybrid metagenomic assembly of OPERA-MS has been developed for use in the study of intestinal metagenomes of patients treated with antibiotics. The mobile elements of the metagenome in the stool sample were successfully assembled with long reading data provided by nanopore sequencing [65]. Among them, new antibiotic resistance has been found with no homology with known sequences, which is of great value in the clinical environment. In summary, nanopore sequencing can identify AMR gene carried by pathogenic bacteria more effectively in a shorter time. This could help clinicians make decisions about customizing antibiotics rather than broad-spectrum ones.

## 3. Description of Disease-Related Microbial Community

Under normal circumstances, each person's body is a rich ecological environment in which the human body and microbial community are finely balanced. However, changes in internal physiological and pathological conditions and external interference can easily upset this balance [66, 67]. In many cases, clinical infections are complex [68], particularly in the lower respiratory and intestinal tracts, where microorganisms are abundant, and the coinfections are not surprising [69]. In the study by Charalampous and collaborators, mixed infections in lower respiratory tract samples were successfully determined by nanopore metagenomics [31].

A common understanding is that microbial diversity is directly related to disease [70, 71]. Metagenomic sequencing is a powerful tool for characterization of microbial community [72]. In the previous studies of metagenomics, Illumina platform has contributed to high accuracy, but its reading of highly fragmented short sequences easily leads to the wrong assembly of genome repeat regions [73]. Nanopore-based sequencers have the ability to overcome this limitation by producing long reads and allowing highly complete genome sequences [74]. In one study, nanopore metagenomic sequencing and Illumina metagenomic sequencing were simultaneously applied to the microbial simulated community, and the results showed that the two platforms submitted similar answers for identification at the level of microbial species classification and abundance [28]. However, for species that are highly similar at the genome level, ONT Platform shows a more accurate species abundance [35].

Targeted sequencing based on 16S RNA gene has also been widely used in microbial classification and diversity research. NGS-based 16S rRNA sequencing strategy only analyzes hypervariable regions (V1-V2 or v3-v4) [75]. Microbial diversity may be largely underestimated. ONT platform provides more accurate identification of bacteria by analyzing the full-length sequence of 16S rRNA gene; at the same time, more accurate taxonomy and clearer developmental science are available [76, 77].

## 4. Conclusions and Future Perspectives

At present, nanopore sequencing has been successfully applied to the monitoring and management of the outbreak of new infectious diseases, identification of drug-resistant pathogenic microorganisms, and identification of disease-related microbial community characteristics, which provides a feasible solution for solving the current epidemic problem. However, it must be acknowledged that this may not be the best option for common pathogens but can improve or fill the gaps in conventional diagnostic tools. And it is important that the personnel to operate should be appropriate for good collection of samples, nucleic acid separation, library preparation, sequencing, and data processing. At the same time, external quality control needs to be established. Moreover, the clinical samples are very complex, there is a high proportion of nucleic acid contamination in the host, and the vulnerable microbial population may be covered [31, 78]. Although strategies to enrich microbial nucleic acids are being developed, the methods of sample preparation still need to be optimized [79, 80]. The accuracy of genome assembly remains a focus of concern. It is therefore urgent to improve the resolution of single base especially for the small genome with high mutation. Of course, the combination of nanopore sequencing and other short reads sequencing is also a potential solution, which can help obtain higher quality genomic information [81–83], but it is believed that, with the further development of the technology, nanopore sequencing alone can provide enough accurate results.

## Figures and Tables

**Table 1:** Advantages and disadvantages of common pathogen detection methods.

Testing methods	Advantages	Disadvantages	Turnaround time	Reference
Culture	Easy to obtain, low cost, simple operation	Sensitivity is affected by antibiotics, limited use in fastidious organisms, time-consuming, unable to detect the gene mutation	Usually takes 5–7 days	[12, 13]

Polymerase chain reaction (PCR) (e.g., direct PCR and multiplex PCR)	Rapid, simple operation, accurate quantification, low cost	Limited to detection of known pathogens, depends on primer design, but the primer is not always effective, unable to detect the gene mutation	One to several hours	[14]

Targeted NGS	High throughput, potential for quantitation highly specific amplification of selected organism types, be able to detect gene mutation	PCR amplification is needed, complicated to operate and long time to result, limited to not covering the entire gene region, depends on hypothesis requiring primers that may not always work, accurate taxonomic identification depends on the quality and completeness of the reference databases	One to several days	[15–17]

Metagenomic NGS	High throughput, no amplification, no bias testing, direct application to clinical samples, potential for discovery of unknown pathogens, be able to detect gene mutation	High cost, long time to result, complicated to operate, vulnerable to human background pollution, difficult to analyze complex genome structure	One to several days	[16, 18]

Nanopore sequencing	Ultralong reads and real-time data, accurate species resolution, direct sequencing of DNA and RNA, high throughput and inexpensive, rapid, portable, and easy to operate, be able to parse complex genome structure, be able to detect gene mutation	Relatively high error rate, quality of sequencing is affected by library quality and sequencing inhibitors	Several hours	[19–21]

**Table 2:** Nanopore sequencing can quickly identify pathogens in various clinical samples.

Sample type	Nucleic acid type	Diagnosis	Sequencing methods	Turnaround time	Pathogen
Blackbird brain tissues	DNA	—	—	—	*Usutu virus* [21]
Whole blood	RNA	—	—	<24 h	*Ebola virus* [20]
Plasma	RNA	—	Metagenomic sequencing	<6 h	*Chikungunya virus* [29]
Whole blood	RNA	—	Metagenomic sequencing	<6 h	*Ebola virus* [29]
Serum	RNA	—	Metagenomic sequencing	<6 h	*Hepatitis C virus* [29]
Cerebrospinal fluid	DNA	Bacterial meningitis	16S sequencing	3 h	*L. monocytogenes*: *S. pneumoniae, P. aeruginosa*: *K. pneumoniae, etc.* [34]
Sputum	DNA	Community-acquired pneumonia	16S sequencing	5 h	*Haemophilus influenzae pneumonia* [30]
Faeces	DNA	Necrotizing enterocolitis	Shotgun metagenomic sequencing	<5 h	*Klebsiella pneumoniae and Enterobacter cloacae* [28]
Sonication fluid	DNA	Prosthetic joint infections	Metagenomic sequencing	—	*Staphylococcus aureus, Cutibacterium acnes, Staphylococcus epidermidis, etc.* [35]

—, not given.
